# Elective laparoscopic cholecystectomy without intraoperative cholangiography: role of preoperative magnetic resonance cholangiopancreatography - a retrospective cohort study

**DOI:** 10.1186/s12893-016-0159-9

**Published:** 2016-07-13

**Authors:** Jinfeng Zang, Yin Yuan, Chi Zhang, Junye Gao

**Affiliations:** Department of Hepatobiliary Surgery, Taizhou People’s Hospital, No. 210, Yingchun Road, Taizhou, 225300 Jiangsu Province China

**Keywords:** Magnetic resonance cholangiopancreatography, Intraoperative cholangiography, Laparoscopic cholecystectomy, Bile duct injury, Common bile duct stones

## Abstract

**Background:**

Laparoscopic cholecystectomy (LC) is the standard treatment for gallbladder diseases. Intraoperative cholangiography (IOC) can reduce biliary complications of LC; however, with the emergence of magnetic resonance cholangiopancreatography (MRCP), IOC nowadays is faced with unprecedented challenge. The purpose of this study is to evaluate whether preoperative MRCP can safely replace IOC during elective LC in terms of retained common bile duct (CBD) stones and bile duct injury (BDI).

**Methods:**

A retrospective study on candidates for elective LC who underwent IOC or preoperative MRCP between January 2009 and December 2014 was conducted.

**Results:**

In the IOC group, 1972 patients underwent LC and 213 required IOC. In the MRCP group, 2268 patients underwent LC and 257 required MRCP. In the IOC group, the rate of retained CBD stones was 0.45 % without IOC and 1.41 % with IOC. In five of 157 patients who underwent IOC, endoscopic retrograde cholangiopancreatography or laparoscopic CBD exploration showed no evidence of CBD stones. In the MRCP group, the rate of retained CBD stones was 0.45 % without MRCP. No patients with normal MRCP findings returned with symptomatic CBD stones during 1-year follow-up. The rate of BDIs was 0.20 % in the IOC group and 0.13 % in the MRCP group.

**Conclusions:**

Selective use of preoperative MRCP is an effective and safe strategy when conducting elective LC to treat gallstones. LC resorting to preoperative MRCP can be performed safely without IOC, with an acceptable rate of retained CBD stones and BDIs.

## Background

Laparoscopic cholecystectomy (LC) is the standard treatment for symptomatic gallbladder diseases. Retained common bile duct (CBD) stones and bile duct injuries (BDIs) are rare but serious complications, closely associated with postoperative morbidity and mortality. Despite the wide variety of examinations, consensus on the diagnostic method ensuring the safety of LC has not been established.

Intraoperative cholangiography (IOC) is an essential procedure during LC, enabling detection of unsuspected CBD stones and anatomical identification of biliary structures [1–5]. Those who recommend IOC maintain that it ensures clearance of CBD stones in the same session and helps prevent BDI. However, IOC is time consuming and has a false-positive rate of 1–3 % [6, 7]. Most surgeons argue that it should be reserved only for patients with strong signs of suspected CBD stones, or in the case of uncertain anatomy. There is also an increasing trend to perform LC without IOC, relying on perioperative screening [8–11].

Magnetic resonance cholangiopancreatography (MRCP) is a noninvasive technique that began to replace endoscopic retrograde cholangiopancreatography (ERCP) for preliminary assessment of suspected biliary obstruction, including CBD stones [12]. Comparatively, MRCP requires shorter examination time, fewer staff, and involves no ionizing radiation. The increasing use of MRCP requires that its role before LC is assessed objectively. No large-sample studies have investigated the role of preoperative MRCP and IOC in the safety of LC. This retrospective study was conducted to evaluate whether preoperative MRCP can safely replace IOC during elective LC in terms of retained CBD stones and BDI.

## Methods

We performed a retrospective study on patients who were candidates for elective LC who underwent IOC or preoperative MRCP to confirm CBD stones at Taizhou People’s Hospital, China between January 2009 and December 2014. The study was approved by our ethics committee, and written informed consent was obtained from each patient. Our hospital provides a comprehensive general surgical service and tertiary care for hepatobiliary surgery, for a population of 5 million people. All operations in our department were performed by consultant surgeons. Follow-up of symptoms or signs of suspected biliary disease was conducted by telephone at 1 week, 1 month and 1 year after LC.

Patients with a history of jaundice, cholangitis or pancreatitis, abnormal liver function tests, ultrasonographic evidence of CBD stones or dilatation underwent IOC or preoperative MRCP for bile duct evaluation. Patients in the IOC group were admitted between January 2009 and December 2011, and patients in the MRCP group were admitted between January 2012 and December 2014. In IOC group, all suspected patients received IOC during LC. In the MRCP group, all suspected patients received MRCP before LC.

Retained CBD stones were discovered within the first year after LC. BDI was any damage to the wall of the main biliary tree detected during LC or diagnosed after surgery as a result of bile leakage or biliary obstruction caused by stenosis. BDIs were classified according to the Strasberg classification.

Patients with malignant biliary obstruction and incomplete postoperative follow-up data were excluded. Medical records and operative reports were reviewed to assess the outcomes of IOC and preoperative MRCP. The results were analyzed using SPSS version 15.0 (SPSS, Chicago, IL, USA). The statistical data were analyzed using the *t* test, Pearson’s *χ*^2^ or Fisher’s exact test. *P* < 0.05 was considered statistically significant.

## Results

### Patient characteristics

The rate of incomplete follow-up was 1.46 % in the IOC group and 1.51 % in the MRCP group. In the IOC group, 1972 patients underwent LC and 213 required IOC. In the MRCP group, 2268 patients underwent LC and 257 required MRCP. There was no difference in the rate of cases undergoing IOC or preoperative MRCP (10.8 % vs 11.3 %; *p* = 0.58). There was no LC-related mortality in either group. Patient characteristics are shown in Table 1. There were no significant differences in age, sex and indications.

**Table 1:** Characteristics of patients receiving IOC and preoperative MRCP

	IOC	MRCP	*p* value
	(*n* = 213)	(*n* = 257)	
Age (yr)	54.3 ± 9.1	55.2 ± 10.0	0.31
Sex (M/F)	97/116	119/138	0.87
Indications			
Positive history	17	21	0.94
Abnormal LFT	185	223	0.98
Abnormal US	94	109	0.71

*LFT* liver function test, *US* ultrasonography

### Retained stones

In the IOC group, 12 patients were readmitted with symptoms and/or signs suggestive of retained CBD stones. In 11 patients, retained CBD stones were confirmed through subsequent ERCP. The 11 cases included three patients in whom IOC was normal. The rate of retained CBD stones was 0.45 % (8/1759) in patients without IOC and 1.41 % (3/213) in patients with IOC. However, in five of 157 patients with positive IOC, subsequent ERCP or laparoscopic CBD exploration showed no evidence of CBD stones.

In the MRCP group, 10 patients without the indication of MRCP before LC were readmitted because of suspected retained CBD stones. In nine patients, retained CBD stones were found by subsequent ERCP. The rate of retained CBD stones was 0.45 % (9/2011) in patients without MRCP. No patients with normal MRCP findings returned with symptomatic CBD stones during 1-year follow-up. Subsequent ERCP or laparoscopic CBD exploration showed no evidence of CBD stones in 12 of 192 patients with positive preoperative MRCP.

**Fig. 1:**
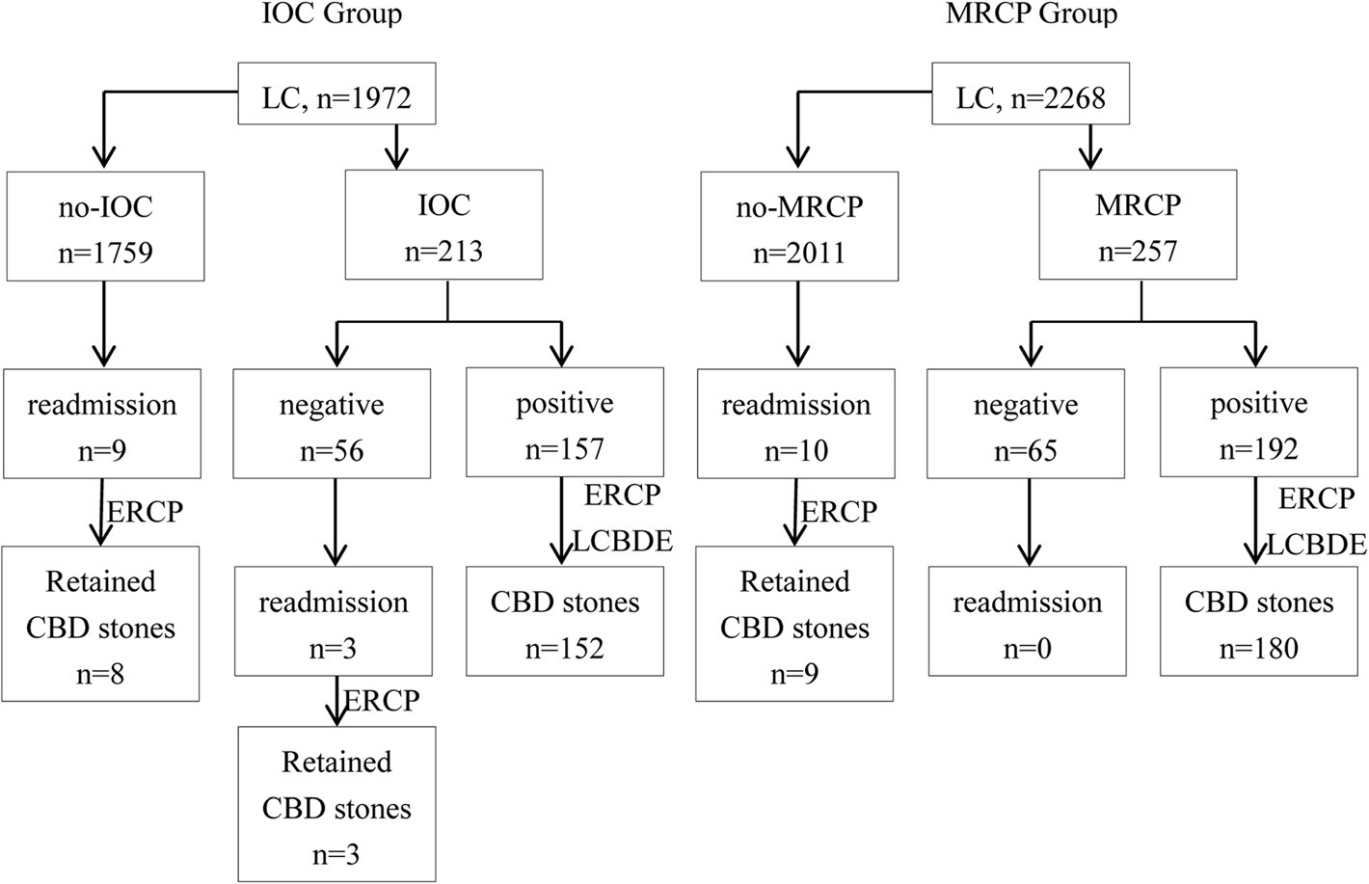
Outcomes of two groups on retained CBD stones. IOC: Intraoperative cholangiography; MRCP: Magnetic resonance cholangiopancreatography; CBD: common bile duct; ERCP: Endoscopic retrograde cholangiopancreatography; LCBDE: Laparoscopic common bile duct exploration

All retained CBD stones were successfully extracted by ERCP. The outcomes of two groups with retained CBD stones are shown in Fig. 1.

### BDI

In the IOC group, four (0.20 %) BDIs were detected: two (0.10 %) CBD injury and two (0.10 %) minor bile leakages. One case of CBD injury (type E1) was diagnosed immediately during LC and underwent choledochocholedochostomy stented by T tube. The other case with CBD injury (type E2) was confirmed postoperatively and received choledochocholedochostomy. The patient eventually underwent Roux-en-Y choledochojejunostomy 8 months after LC because of anastomotic stricture. No further late complications occurred in this case. Two minor bile leakages (one type A and the other type D) were diagnosed early after LC and cured through percutaneous drainage of intra-abdominal bile collections.

In the MRCP group, three (0.13 %) BDIs occurred: one (0.04 %) CBD injury and two (0.09 %) minor bile leakages. The case with CBD injury (type E1) was diagnosed immediately during LC and operations were converted to laparotomy. The continuity of transected CBD was restored by choledochocholedochostomy and secured by T tube. Two minor bile leakages were diagnosed in the early postoperative period. One (type D) was treated with endoscopic nasobiliary drainage and percutaneous drainage of intra-abdominal bile collections, and the other (type A) only with percutaneous drainage. These patients recovered without long-term complications. There was no significant difference in the incidence of BDIs (*p* = 0.32).

## Discussion

LC is the standard surgical treatment for benign gallbladder diseases. There is debate surrounding imaging during LC. Appropriate imaging techniques can detect gallbladder diseases and exhibit the biliary tree precisely. Imaging should be cost-effective and incur few physical injuries. Clinical examination, laboratory tests and ultrasonography before LC have low costs and are available in most medical centers. This strategy has been used to screen suspected cases with biliary disease. However, it sometimes fails to recognize choledocholithiasis and does not show the biliary tree distinctly.

IOC was first described in 1932 and is a major method for diagnosis of choledocolithiasis during cholecystectomy [13]. Although the primary objective of IOC is diagnosis of bile duct stones, it can also diagnose or prevent iatrogenic biliary injuries. Therefore, some advocate that IOC should be adopted in all LCs because it reveals the anatomy of the biliary tract and detects CBD stones. Nevertheless, only a few institutions still perform routine IOC during LC and some even perform LC without IOC. An average 750 000 cholecystectomies are performed in the US annually, and only 27 % of surgeons perform IOC routinely, while the rest use it only in cases with suspected biliary stones or iatrogenic injury [14].

According to sensitivity, specificity and predictive ability, IOC is a safe and accurate method for detection of bile duct stones. In a meta-analysis involving 4209 patients without preoperative suspicion of choledocholithiasis, IOC was positive in 170 (4 %) cases [15]. The false-positive rate was 0.8 % (34 cases), which is lower than the reported rate (1.6 %). In the same study, 32 (0.6 %) cases from 5179 preoperatively unsuspected LCs without IOC developed complications due to residual stones after follow-up. In the era of minimally invasive surgery, diagnostic approaches have emerged in an attempt to incur fewer traumas and permit rapid postoperative recovery [16]. However, IOC is an invasive diagnostic method that may lengthen the surgical procedure under anesthesia and cause BDI during attempts to cannulate a narrow and short cystic duct, or involuntarily cannulate the CBD [9, 17]. It can also have false-positive results in 1–3 % of cases, resulting in unnecessary biliary tract exploration.

The role of IOC in prevention and management of BDI remains controversial [18–20]. The rate of iatrogenic BDIs is 0.4–0.6 % in LC compared to the open procedure (0.2–0.3 %). Although iatrogenic injuries are more readily recognized by IOC, some studies found no difference in the incidence of BDIs between routine and selective IOC, and no association between anatomical anomalies and iatrogenic injuries. Some studies have demonstrated no benefit in preventing BDI using IOC. An Italian study of 56 591 LCs performed during 1998–2000 reported a BDI incidence of 0.42 % [21]. There was no significant difference when IOC was performed routinely or selectively (0.32 % vs 0.43 %). In a multicenter retrospective study of 2714 cases, five (0.18 %) had major BDIs requiring surgical repair [22]. Postoperative bile leakage was encountered in seven cases (0.26 %). The authors concluded that LC can be performed safely without IOC, with acceptable rates of biliary complications, provided that there is proper detection of silent CBD stones and postoperative ERCP is available. A retrospective cohort study comprising all Texas Medicare patients from 2000 to 2009 compared IOC during LC from multivariate logistic regression models with instrumental variable analyses [23]. The BDI rate was 0.21 % among 37 533 patients with IOC and 0.36 % among 55 399 patients without IOC. However, the association between LC performed without IOC and BDI was no longer significant when confounding was controlled with instrumental variable analysis. In contrast, another systematic review found a protective effect of routine IOC on BDI during LC. The study from Argentina of 11 423 consecutive LCs during 1991–2012 showed that routine IOC in LC was associated with a low incidence of BDI, and facilitated detection and repair during the same surgical procedure with a good outcome [24].

Even with experienced surgeons who perform IOC routinely, the rate of iatrogenic BDI is not zero. Therefore, the use of routine IOC for prevention of BDI is debatable. In the policy of LC without IOC, Ammori thought that LC could be performed without IOC as selective preoperative MRCP and ERCP detected choledocholithiasis effectively and careful operative technique avoided duct injury safely [9]. In our study, the rate of BDI was 0.20 % in the IOC group and 0.13 % in the MRCP group. The rate of BDI was low in both groups and there was no significant difference between preoperative MRCP and IOC. Owing to careful training and strict laparoscopic practice, all surgeons in our departments are on the stable phase of the LC learning curve. We consider that effective prevention of BDI during LC is not due to imaging modality but rather careful operative technique. According to Sanjay’s practice [25], the “critical view of safety” technique in LC has been applied routinely in our department since 2011. Although routine IOC is not recommended in LC in most current reports, we agree that IOC allows direct identification of bile duct anatomy and early diagnosis of BDI, and is used in cases of uncertain anatomy.

MRCP visualization of fluid in the biliary tract without contrast was first introduced in 1986 for diagnosis of biliary disease, to demonstrate the anatomy of the dilated bile ducts and the location of the obstruction [26]. There is no exposure to ionizing radiation when patients receive MR examination. Nowadays, MRCP is a reliable and noninvasive method for detection or exclusion of CBD stones before LC. Chang et al. showed that no patient whose MRCP showed a clear CBD returned with symptomatic stones during 1 year follow-up, which suggests a high negative predictive value for MRCP [27].

In our study, CBD stones that were missed by ultrasonography were detected by MRCP. The smallest stone detected with MRCP was 3 mm in diameter, and the smallest detected by IOC was 4 mm. Three patients who were free of CBD stones by IOC were diagnosed with CBD stones at follow-up. When the cholangiography data were traced, the retained stones, with diameter <5 mm, were mostly located in clearly dilated ducts (15 mm diameter). Therefore, the efficacy of IOC for detection of small stones in dilated ducts might be lower than that of MRCP. Routine MRCP before LC is helpful to reduce the incidence of postoperative complications [12]. MRCP can be performed in patients undergoing elective LC to investigate possible variants of cystic duct. A study reported by Ausch et al. showed that preoperative MRCP disclosed 27 of 462 patients (6 %) with anatomical variants in the cystic duct and its confluence, improving the safety of LC [28].

Compared to IOC, MRCP has another advantage of devising therapeutic modality beforehand. When IOC in LC confirms the CBD stones in a nondilated duct, intraoperative ERCP might be the best choice because laparoscopic CBD exploration is difficult with small-diameter CBDs. Although ERCP can be performed by surgeons in our operating room, we do not recommend this unconventional practice [29]. MRCP is more beneficial than IOC for selecting the method of CBD stone extraction. Although the negative prediction of CBD stones with MRCP is more accurate, the false-positive rate of MRCP was twice that of IOC. Therefore, more attention should be paid to unnecessary bile duct exploration when preoperative MRCP is performed during LC to diagnose CBD stones.

In addition to medical considerations, surgeons must also consider the costs when they determine clinic examinations. Although the costs of diagnostic modalities differ markedly among countries and healthcare systems, combination of transabdominal ultrasound and serum biochemical tests is a preliminary predictor of CBD stones. Although MRCP requires no special skills for surgeons, the routine use of it is not usually recommended due to economic considerations. For cost-effective diagnosis and management of asymptomatic choledocholithiasis, Epelboym et al. showed that LC with routine IOC was more cost-effective than universal MRCP and ERCP in excluding CBD stones [30].

## Conclusions

The results of the present study show that selective use of preoperative MRCP is an effective and safe strategy when conducting elective LC to treat gallstones. This imaging modality might replace IOC and allow LC to be performed safely with an acceptable rate of retained CBD stones and BDIs.

## Abbreviations

BDI, bile duct injury; IOC, intraoperative cholangiography; LC, laparoscopic cholecystectomy; MRCP, magnetic resonance cholangiopancreatography
